# Morgagni hernia: an uncommon pathology in adults

**DOI:** 10.1093/jscr/rjac597

**Published:** 2022-12-30

**Authors:** Karnan Rajkumar, Sayali Kulkarni, Toghrul Talishinskiy

**Affiliations:** Department of General Surgery, St. Joseph’s University Medical Center, Paterson, NJ, USA; Department of General Surgery, St. Joseph’s University Medical Center, Paterson, NJ, USA; Department of General Surgery, St. Joseph’s University Medical Center, Paterson, NJ, USA

**Keywords:** Morgagni hernia, Bochdalek hernia, colonic dilation, mesh placement, laparoscopic repair

## Abstract

Typically, diaphragmatic hernias occur as congenital defects and are considered a rare presentation when seen in adults. They occur as developmental defects and stem from embryonic components of the diaphragm not fusing completely. There are two types of diaphragmatic hernias, classified based on the location of herniation through the diaphragmatic defect. Bochdalek hernias present as defects in the left postero-lateral diaphragm, whereas Morgagni hernias present as anterior defects of the diaphragm. The more common defect of the two are Bochdalek hernias making Morgagni hernias a rare presentation. This case describes the presentation of a hernia through an anterior defect in the diaphragm, otherwise classified as a Morgagni hernia.

## INTRODUCTION

Diaphragmatic hernias can be broken down into congenital versus acquired. There are three types of congenital diaphragmatic hernias that include: Bochdalek hernia, Hiatal hernia and Morgagni hernias. The most common of the three are Bochdalek hernias, which are seen in about 95% of cases. Acquired Morgagni hernias constitute only 2–4% of congenital diaphragmatic hernias and symptomatic adult cases are extremely rare [1]. For this reason, it makes our case of Morgagni hernia discovered in adulthood a rare presentation.

This case describes a patient who presented with weakness in the diaphragm at the anterior portion causing protrusion of bowel through the diaphragm. These defects are most often seen in neonates; however, this report details the case of an adult patient that underwent laparoscopic repair of a diaphragmatic hernia with mesh placement. Although Morgagni hernias occur because of a congenital diaphragmatic defect, conditions such as pregnancy, trauma, chronic cough, obesity and constipation may increase the intra-abdominal pressure, and predispose to the development of the condition [1]. Our patient had many chronic problems that could have predisposed her to diaphragmatic herniation. These included chronic obstructive pulmonary disease (COPD) leading to acute respiratory insufficiency, chronic combined systolic and diastolic heart failure, coronary artery disease, paroxysmal atrial fibrillation, hypothyroidism, hyponatremia and malnutrition.

**Figure 1:**
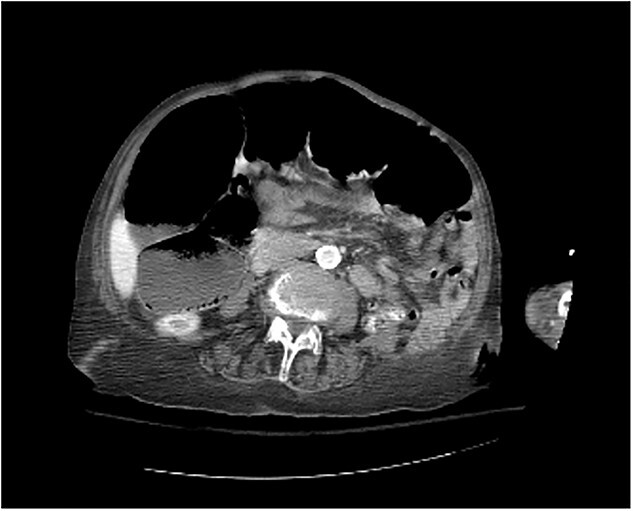
Colonic dilation seen on CT abdomen and pelvis with contrast.

Upon presentation, she did have signs indicative of gastrointestinal and respiratory complications. She presented with coffee-ground emesis, poor appetite and abdominal distension. She also presented with respiratory distress evidenced by increased respirations and shortness of breath. Most cases of diaphragmatic hernia go unnoticed as they are often asymptomatic; however, this patient had signs of bowel incarceration leading to bowel obstruction necessitating surgical repair. Although the patient had extensive preexisting medical comorbidities, the indication for surgery was high because of the risk of bowel strangulation from the persistent colonic dilation (Fig. 1).

## CASE REPORT

An 84-year-old female with a past medical history of COPD, atrial fibrillation, macular degeneration, coronary artery disease, microcytic anemia, chronic combined systolic and diastolic heart failure, history of nicotine use, severe malnutrition, steroid-induced hyperglycemia and acute on chronic respiratory failure presented to the ED with nausea and vomiting. The patient reported that the vomiting was triggered from coffee intake. She recently was admitted at the beginning of July for persistent diarrhea. She was discharged with Imodium and told to follow-up with her gastroenterologist after the diarrhea resolves, but she reported feeling unwell ever since her last visit. She had been having persistent nausea, vomiting and the inability to tolerate a diet for the past few days. She reported her last bowel movement was 3 days ago. She denied fever, chills and chest pain but complained of significant diffuse abdominal pain.

**Figure 2:**
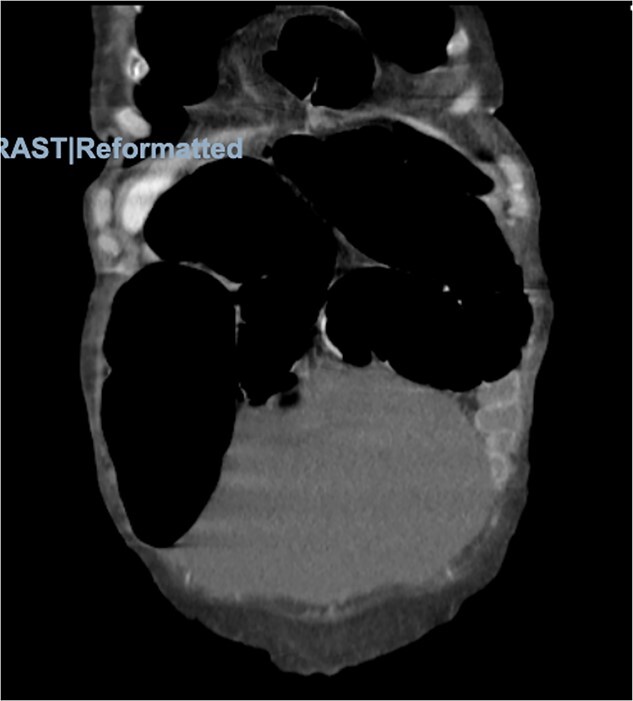
Morgagni hernia seen on CT abdomen and pelvis with contrast.

**Figure 3:**
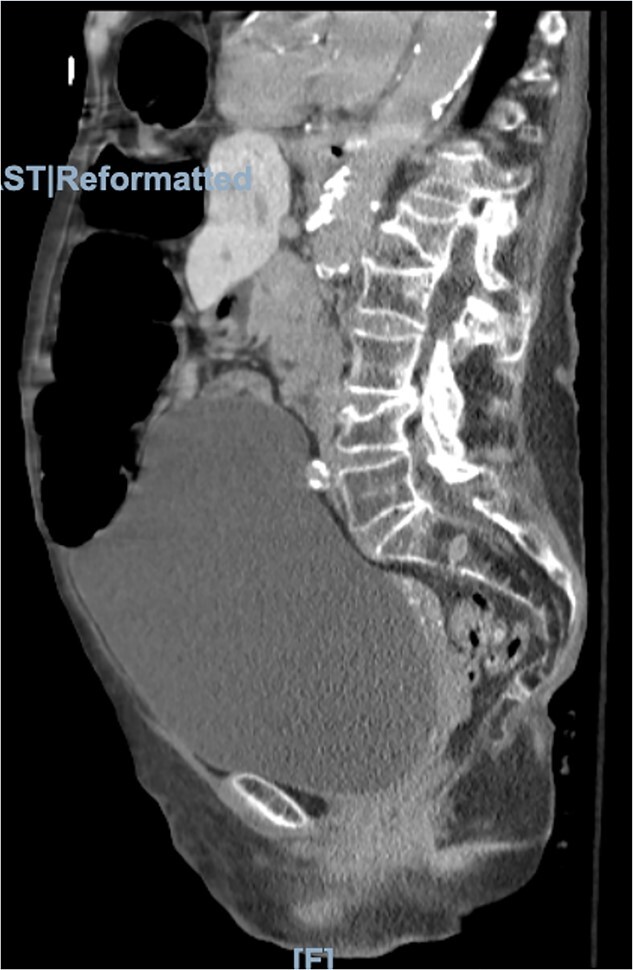
Morgagni hernia seen on CT abdomen and pelvis with contrast (sagittal view).

Physical examination was significant for right upper quadrant and epigastric tenderness with a mildly distended abdomen. On admission, the patient had a leukocytosis of 16.2. Imaging included computed tomography (CT) of the abdomen and pelvis showing the obstruction of the colon suspected because of a loop of colon herniated into the lower anterior chest, between the lower aspect of the sternum and the left heart border with mild mass-effect on the heart (Figs 2 and 3).

**Figure 4:**
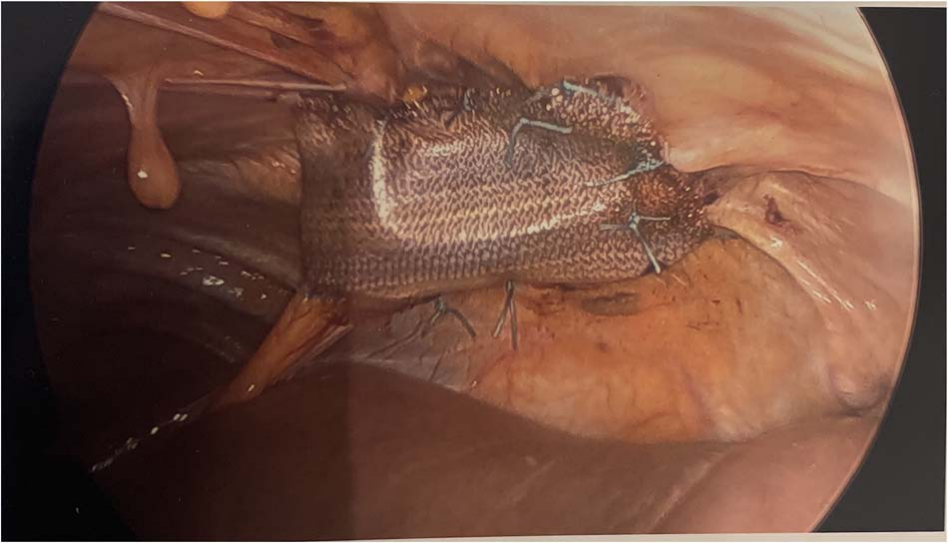
Intraoperative photograph showing Phasix mesh secured across the hernia.

The patient was taken for a diagnostic laparoscopy with mesh repair of the diaphragmatic hernia. Upon initial visualization via diagnostic laparoscopy, the colon was noted to have spontaneously reduced from the diaphragmatic hernia site. The antimesenteric border of the small portion of the colon was noted to appear ecchymotic. The colon was noted to be very dilated suggesting symptoms of obstruction. The actual diaphragmatic hernia defect was noted to be lateral to the falciform ligament measuring ~ 4 cm wide by 2 cm in height. A 7 × 5 cm Phasix mesh was secured across the hernia defect using 3-0 Ethibond suture in an interrupted fashion. Care was taken to ensure the mesh extended well beyond the visualized defect (Fig. 4).

## DISCUSSION

Of the different types of congenital diaphragmatic hernias, Morgagni hernias are the rarest of them all. Presentation during adulthood is uncommon. The majority of the patients with Morgagni hernias are asymptomatic and diagnosis is usually made from routine chest X-ray. Some patients may present with nonspecific cardiac, respiratory or gastrointestinal symptoms. Rarely, acute abdominal or thoracic symptoms caused by obstruction and strangulation of the bowel may lead to further investigation and diagnosis.

Morgagni hernias can be repaired via an abdominal approach (laparotomy or laparoscopically) or transthoracic approach [2]. In our case, we proceeded with the laparoscopic approach. Initially, as with any hernia, the hernia contents are reduced, and then following reduction, the diaphragmatic defect is repaired. Repair of the diaphragmatic defect can be achieved with the use of sutures for primary closure or with the use of a synthetic or biological mesh to cover the defect. Mesh repair is indicated when the defect is too large for primary closure [3]. Currently, there is no clear consensus for when to use primary closure versus mesh repair, or whether transabdominal or transthoracic surgical approach is superior. According to a series of 36 patients undergoing laparotomy versus thoracotomy, surgeons were able to perform a successful repair in the entirety of their Morgagni hernia cases without the use of mesh and without recurrence [4]. However, this has varied from case to case as it depends on the amount of tissue that has been lost because of the herniation and the thinning of the diaphragm. In our case, the hernia defect measured 4 × 2 cm, which indicated a large fascial defect necessitating closure with mesh. Some surgeons advocate for the use of a polypropylene mesh, whereas others have used intermittent or continuous nonabsorbable suture to close the defect [5]. Therefore, the only definitive criteria for surgical repair is the size of the hernial defect. The decision to perform a mesh versus primary repair is surgeon dependent as there is currently a lack of data because of the rarity of these cases to establish objective guidelines. Case presentations such as these can help showcase that a patient with a rare diagnosis may present with unusual symptoms complicating a straightforward diagnosis.

Signs of obstruction and impending danger of bowel necrosis can necessitate emergent operative intervention. Diagnosis can be difficult and a missed diagnosis can lead to life threatening complications such as extensive bowel resection and sepsis [6]. Our patient had signs of colonic dilation, bowel obstruction and ecchymosis of the colon. Although these herniations are rare, the life-threatening complications associated with them are severe necessitating prompt diagnosis.

## CONCLUSION

Further education about diaphragmatic hernias is necessary to identify the risk factors, signs, symptoms and varied presentations to definitively diagnose and move forward with surgical repair in a timely manner. Surgical techniques should be highlighted to establish set guidelines in repairing these hernial defects. All these factors play a major role in the management of diaphragmatic hernias as it can prevent unnecessary complications and lead to timely diagnosis.

## CONFLICT OF INTEREST STATEMENT

None declared.

## FUNDING

None.

## ETHICAL CONSENT

Patient permission granted for the publication of the case.
